# Supplementary material for: Joint epigenome profiling reveals cell-type-specific gene regulatory programmes in human cortical organoids
Source: Nat Cell Biol. 2023 Nov 23;25(12):1873–83. doi: 10.1038/s41556-023-01296-5 (PMC10709149; doi:10.1038/s41556-023-01296-5)
Supplement: Supplementary file 1 — Reporting Summary [file 41556_2023_1296_MOESM1_ESM.pdf]

nature portfolio

Corresponding author(s): Boyan Bonev

Last updated by author(s): Aug 28, 2023

Reporting Summary

Nature Portfolio wishes to improve the reproducibility of the work that we publish. This form provides structure for consistency and transparency in reporting. For further information on Nature Portfolio policies, see our Editorial Policies and the Editorial Policy Checklist.

Statistics

For all statistical analyses, confirm that the following items are present in the figure legend, table legend, main text, or Methods section.

n/a	Confirmed	
☐	☒	The exact sample size (*n*) for each experimental group/condition, given as a discrete number and unit of measurement
☐	☒	A statement on whether measurements were taken from distinct samples or whether the same sample was measured repeatedly
☐	☒	The statistical test(s) used AND whether they are one- or two-sided<br>*Only common tests should be described solely by name; describe more complex techniques in the Methods section.*
☐	☒	A description of all covariates tested
☐	☒	A description of any assumptions or corrections, such as tests of normality and adjustment for multiple comparisons
☐	☒	A full description of the statistical parameters including central tendency (e.g. means) or other basic estimates (e.g. regression coefficient) AND variation (e.g. standard deviation) or associated estimates of uncertainty (e.g. confidence intervals)
☐	☒	For null hypothesis testing, the test statistic (e.g. *F*, *t*, *r*) with confidence intervals, effect sizes, degrees of freedom and *P* value noted<br>*Give P values as exact values whenever suitable.*
☒	☐	For Bayesian analysis, information on the choice of priors and Markov chain Monte Carlo settings
☒	☐	For hierarchical and complex designs, identification of the appropriate level for tests and full reporting of outcomes
☐	☒	Estimates of effect sizes (e.g. Cohen's *d*, Pearson's *r*), indicating how they were calculated

Our web collection on statistics for biologists contains articles on many of the points above.

Software and code

Policy information about availability of computer code

Data collection	No software was used for data collection
Data analysis	imageJ (2.1.0), FlowJo (10.8.1), FACSDiva (8.0.1), MPRAflow (2.2), MPRAanalyze (1.11), HiCRep (1.12.2), Juicebox (1.19), chromvar (1.6), JuiceMe (1.5.6), Methylkit (1.10), gNOMeHMM (0.2.1), Shaman (2.0), SeqPlots (1.22.2), modified TAURUS-MH pipeline , R, ENCODE ChIP–seq and ATAC-seq pipeline (2.3), monaLisa (0.1.5). All code as well as a list of all used packages including version numbers is available at: https://github.com/BonevLab/NoackVangelisti_2023.

For manuscripts utilizing custom algorithms or software that are central to the research but not yet described in published literature, software must be made available to editors and reviewers. We strongly encourage code deposition in a community repository (e.g. GitHub). See the Nature Portfolio guidelines for submitting code & software for further information.

Data

Policy information about availability of data

All manuscripts must include a data availability statement. This statement should provide the following information, where applicable:

- Accession codes, unique identifiers, or web links for publicly available datasets
- A description of any restrictions on data availability
- For clinical datasets or third party data, please ensure that the statement adheres to our policy

All raw and processed sequencing data are available in the Gene Expression Omnibus (GEO) repository: GSE211736.Previously published data that were re-analysed

here are available under accession codes: RNA-seq (GSE19608425, GSE961075), Methyl-HiC (GSE119171), Methyl-3C (GSE124391), WGBS (GSE112520), Hi-C (GSE96107), ATAC-seq (GSE113592), H3K27ac ChIP-seq (ENCSR000CGQ), DHS (GSE51336), MNase-seq (GSE58101), Ctcf ChIP-seq (GSE96107)5, CTCF ChIP-seq (GSE116825), Smc1 ChIP-seq (GSE22557), Neurog2 ChIP-seq (GSE63621), Neurod2 ChIP-seq (GSE67539), Micro-C (GSE130275), Nrf1 ChIP-seq (GSE67867).

Human research participants

Policy information about studies involving human research participants and Sex and Gender in Research.

Reporting on sex and gender	N/A
Population characteristics	N/A
Recruitment	N/A
Ethics oversight	N/A

Note that full information on the approval of the study protocol must also be provided in the manuscript.

Field-specific reporting

Please select the one below that is the best fit for your research. If you are not sure, read the appropriate sections before making your selection.

☒ Life sciences ☐ Behavioural & social sciences ☐ Ecological, evolutionary & environmental sciences

For a reference copy of the document with all sections, see nature.com/documents/nr-reporting-summary-flat.pdf

Life sciences study design

All studies must disclose on these points even when the disclosure is negative.

Sample size	Sample sizes for all data types are provided in the Supplementary Data Table 2. Sample sizes for 3DRAM-seq and MPRA were chosen based upon the ability to get representative data described based upon analogous studies in the field and to ensure replication of the results with affordable cost.
Data exclusions	No data was excluded from the analysis.
Replication	3DRAM-seq in mESCs was performed in three biological replicates. 3DRAM-seq in cortical organoids was performed in biological duplicates. Cell-type specific MPRA in organoids was performed in two biological replicates. All attempts of replications were successful.
Randomization	For the 3DRAM-seq and MPRA, there was no randomization performed as they do not involve multiple study groups. For the mouse vs human FBXO32 enhancer electroporation experiments, organoids were assigned to the mouse (mE2-Fbxo32) or human (E2-FBXO32) group based on the construct used during the procedure.
Blinding	The investigators were not blinded to the group as no human subjects were involved and no subjective measurements were taken.

Reporting for specific materials, systems and methods

We require information from authors about some types of materials, experimental systems and methods used in many studies. Here, indicate whether each material, system or method listed is relevant to your study. If you are not sure if a list item applies to your research, read the appropriate section before selecting a response.

Materials & experimental systems

n/a	Involved in the study	
☐	☒	Antibodies
☐	☒	Eukaryotic cell lines
☒	☐	Palaeontology and archaeology
☒	☐	Animals and other organisms
☒	☐	Clinical data
☒	☐	Dual use research of concern

Methods

n/a	Involved in the study	
☒	☐	ChIP-seq
☐	☒	Flow cytometry
☒	☐	MRI-based neuroimaging

Antibodies

Antibodies used	For flow-cytometry following antibodies were used: SOX2-PE (BD Biosciences, Cat. N.: 562195, clone O30-678, dilution 1:20), PAX6-AlexaFluor488 (BD Biosciences, Cat. N.: 561664, clone O18-1330, dilution 1:40) and EOMES-eFluor660 (ThermoFisher, Cat. N.: 50-4877-41, clone WD1928, dilution 1:20). For immunohistochemistry the following antibodies were used: anti-PAX6 (Biolegend, Cat. N.: 901301, clone Poly19013, dilution 1:100), anti-EOMES (R&D Systems, Cat. N.: AF6166, Polyclonal Sheep IgG, dilution 1:150), donkey anti-rabbit-A555 (Thermo Scientific, Cat. N.: A32794,dilution 1:1000), donkey anti-sheep-A488(Thermo Scientific, Cat. N.: A11015, dilution 1:1000), goat anti-Chicken A488 (Thermo Scientific, Cat. N.: A11039, Polyclonal Goat IgG, dilution 1:1000), donkey anti-goat APlus 555 (Thermo Scientific, Cat. N.: A32816, dilution 1:1000), anti-RFP (Rockland, Cat. N.: 200-101-379, Polyclonal Goat IgG, dilution 1:1000), anti-GFP (Abcam, Cat. N.: ab13970, Chicken polyclonal IgY, dilution 1:1000), anti-Oct3/4 A488 (BD Biosciences, Cat. N.: 560253, clone 40/Oct-3, dilution 1:33), PE anti-Sox2 (BD Biosciences, Cat. N.: 560291, clone 245610, dilution 1:50), V450-SSEA-4 (BD Biosciences, Cat. N.: 561156 clone MC813-70, dilution 1:67), Alexa Fluor 647 anti Tra-1-60 (BD Biosciences, Cat. N.: 560122, clone TRA-1-60, dilution 1:67), SOX17 primary antibody (Abcam, Cat, N.: ab84990, clone OTI3B10, dilution 1:200), Alexa Fluor 488 goat anti-mouse IgG (ThermoFisher, Cat. N.: A11001, dilution 1:200).
Validation	Antibodies were validated by the respective supplier:<br>SOX2-PE (https://www.bdbiosciences.com/ko-kr/products/reagents/flow-cytometry-reagents/research-reagents/single-color-antibodies-ruo/pe-mouse-anti-sox2.562195)<br>PAX6-AlexaFluor488: (https://www.bdbiosciences.com/en-nz/products/reagents/flow-cytometry-reagents/research-reagents/single-color-antibodies-ruo/alexa-fluor-488-mouse-anti-human-pax-6.561664)<br>EOMES-eFluor660: (https://www.thermofisher.com/antibody/product/EOMES-Antibody-clone-WD1928-Monoclonal/50-4877-42)<br>anti-PAX6 (https://www.biolegend.com/fr-fr/products/purified-anti-pax-6-antibody-11511)<br>anti-EOMES (https://www.rndsystems.com/products/human-eomes-antibody_af6166)<br>Additionally, RNA-seq (Figure 4C) or qPCR (Extended Date Figure 7D) of FAC-sorted cell populations was performed.

Eukaryotic cell lines

Policy information about cell lines and Sex and Gender in Research

Cell line source(s)	mESC E14TG2a was obtained from ATCC (Cat. N.: CRL-1821). The hiPS cells human cortical organoid generation were kindly provided by the Helmholtz Zentrum München iPSC core facility (HMGU1 hiPSC line). For electroporation of human organoids the hIPS cell line CRTDi004-A was used.
Authentication	E14TG2a mESC or HMGU1 hIPS line were authenticated by ATCC (https://www.atcc.org/products/crl-1821) or the Helmholtz Zentrum München iPSC core facility (https://www.helmholtz-muenchen.de/fileadmin/IPSC/PDF/HMGU1_datasheet_AP_ER_18052018.pdf), respectively. CRTDi004-A were authenticated by the CRTD Stem Cell Engineering Facility at Technische Universität Dresden as described in the manuscript (https://hpscreg.eu/cell-line/CRTDi004-A).
Mycoplasma contamination	All cell lines were tested negative for mycoplasma contamination.
Commonly misidentified lines<br>(See ICLAC register)	No misidentified lines were used.

Flow Cytometry

Plots

Confirm that:

☒ The axis labels state the marker and fluorochrome used (e.g. CD4-FITC).

☒ The axis scales are clearly visible. Include numbers along axes only for bottom left plot of group (a 'group' is an analysis of identical markers).

☒ All plots are contour plots with outliers or pseudocolor plots.

☒ A numerical value for number of cells or percentage (with statistics) is provided.

Methodology

Sample preparation	Organoids were dissociated using a papain-based neural dissociation kit (Miltenyi Biotec, Cat. N: 130-092-628) according to the manufacturer protocol with minor modifications. Dissociated cells were fixed in 1% Formaldehyde, quenched with 0.2M Glycine followed by permeabilization using 0.1% Saponin (Sigma-Aldrich, Cat. N: SAE0073). Cells were stained for 1h at 4C̈ for SOX2-PE (1:20; BD Biosciences, Cat. N.: 562195), PAX6-AlexaFluor488 (1:40; BD Biosciences, Cat. N.: 561664) and EOMES-eFluor660 (ThermoFisher, Cat. N.: 50-4877-41) in staining buffer containing 0.1 % Saponin, 0.5x cOmplete, EDTA-free Protease Inhibitor Cocktail (Roche, Cat.N.: 11873580001) and 1:25 RNAse Inhibitor (Promega, Cat. N.: N261A). Stained cells were washed 4 times including one wash with washing buffer containing DAPI (1:1000; ThermoFisher, Cat. N: 62248). Cells were passed through a 40µM cell strainer and immediately FAC-sorted.
Instrument	Cell sorting was carried out on a FACSAria Fusion (BD Biosciences; laser: 405nm, 488nm, 561nm, 640nm) or a FACSAria III (BD Biosciences; laser: 405nm, 488nm, 561nm, 633nm) using a 100µm nozzle.
Software	BD FACSDiva

March 2021

Cell population abundance	Abundance of relevant cell populations are shown in Extended Data Figure 4A and 7C. Purity of FAC-sorted cells were determined by RNA-seq (Figure 4C) or qPCR of relevant marker genes (Extended Date Figure 7D).
Gating strategy	Singlets were selected using forward and side scatter followed by the identification of cells in G0/G1 by genomic content based on DAPI staining (only for 3DRAM-seq). These cells where further divided into SOX2-/EOMES+ for hIPC and SOX2+/PAX6+/EOMES- for hRGC as well as triple negative cells (only MPRA).

☒ Tick this box to confirm that a figure exemplifying the gating strategy is provided in the Supplementary Information.
